# Characteristics of EEG power spectra involved in the proficiency of motor learning

**DOI:** 10.3389/fnins.2023.1094658

**Published:** 2023-07-10

**Authors:** Hiroyuki Hamada, Wen Wen, Tsubasa Kawasaki, Atsushi Yamashita, Hajime Asama

**Affiliations:** ^1^Department of Precision Engineering, The University of Tokyo, Bunkyo-ku, Tokyo, Japan; ^2^Research into Artifacts, Center for Engineering, The University of Tokyo, Bunkyo-ku, Tokyo, Japan; ^3^Department of Psychology, Rikkyo University, Niiza, Saitama, Japan; ^4^Department of Physical Therapy, School of Health Sciences, Tokyo International University, Kawagoe, Saitama, Japan; ^5^Department of Human and Engineered Environmental Studies, Graduate School of Frontier Sciences, The University of Tokyo, Kashiwa, Chiba, Japan

**Keywords:** motor learning, electroencephalogram (EEG), power spectra, neural oscillations, rehabilitation, gamma band, neuromodulation, neurorehabilitation

## Abstract

Neuromodulation techniques for modulating brain activity can affect performance in a variety of behaviors. Techniques including transcranial alternating current stimulation and random noise stimulation can modulate neural oscillations. However, the intervention effect of neuromodulation approaches on motor learning is poor, partly because the electroencephalography (EEG) power spectra associated with the motor learning process has not yet been fully elucidated. Therefore, we investigated the characteristics of EEG power spectra in the process of motor learning in 15 right-handed healthy participants (5 females; mean age = 22.8 ± 3.0 years). The motor task was a ball-rotation task in which participants rotated two balls in the palm of their left hand. Participants performed a pre-test, the motor learning tasks, and a post-test. In the motor learning tasks, twenty 60 s trials were performed in the clockwise (CW) direction. Before and after the motor learning tasks, CW and counterclockwise (CCW; control condition) tasks were performed for 60 s each as pre- and post-tests. Therefore, CW direction was set as a motor learning task, while CCW was a test-only control task. EEG was recorded during the tests and tasks, and the power spectra in the alpha, beta, and gamma oscillations were calculated and compared between pre- and post-tests. The results showed that in the CW post-test, the power of the gamma band in the left parietal areas and the right frontal area was significantly higher than in the pre-test. In the CCW, there was no significant difference in each band at each area between the pre- and post-tests. Our findings reveal the characteristics of the EEG spectra related to the motor learning process. These results may help to establish more effective neuromodulation approaches to modifying neural oscillations in motor learning, including in rehabilitation fields.

## 1. Introduction

Motor learning is defined as “a set of (internal) processes associated with practice or experience leading to relatively permanent changes in the capability for responding” (Schmidt, 1988) and results from brain activity and synaptic organization (Macintosh et al., 2007; Xu et al., 2009; Lee et al., 2013; Kim et al., 2015). These processes are thought to be associated with the rehabilitation process aimed at reacquisition of activities of daily living in hemiplegic patients after stroke (Krakauer, 2006; Eberle et al., 2021). Therefore, understanding the mechanisms of motor learning and developing methods to facilitate the process have potential applications in the rehabilitation field.

In recent years, neuromodulation techniques, which are methods to modulate brain activity, have been drawing attention. These techniques are intended to temporarily enhance or inhibit the excitability of brain activity by applying magnetic or electrical stimulation over the scalp, thereby affecting performance (Herrmann et al., 2013; Kang et al., 2016). Of the neuromodulation methods, transcranial alternating current stimulation (tACS) and transcranial random noise stimulation (tRNS) are approaches that modulate neural oscillations of brain regions by applying weak alternating current electrical stimulation of a specified frequency or a specified interval of frequency between electrodes placed on the scalp (Antal et al., 2008; Antal and Herrmann, 2016).

The interventions correspond to the characteristics of the brain activity in the task. In relation to motor learning, a previous study using fMRI reported that the frontal and parietal regions, visual cortex, and temporal cortex were involved in correcting errors to achieve a fast time constant during a motor learning (visuomotor adaptation) task, and increased activity in the lateral parietal lobe and cerebellum was observed for intermediate and slower time constants (Kim et al., 2015). Also, the left lateral parietal region has been shown to be strongly involved in a visuomotor adaptation task with online movement corrections (Mutha et al., 2011). In addition, it is known that neural oscillation activity (gamma band) in the primary motor cortex is increased in relation to motor control based on sensory feedback during human movement and motor learning (Nowak et al., 2018), that beta band activity in the motor cortex is increased during motor observation in healthy subjects and stroke patients (Zhu et al., 2019; Hsieh et al., 2021), and that activity of the alfa and beta bands of the sensorimotor during observation, preparation and execution of a motor task is involved in individual differences in motor skills (Nakano et al., 2013). In resting-state brain activity, changes in the network between regions in the alpha band affect offline motor learning (Manuel et al., 2018), and the strength of connections in the beta band relates to adaptive motor learning ability (Özdenizci et al., 2017). Furthermore, a previous study reported a reduction in beta desynchronization after motor learning in a foot motor task compared to before the learning (Gehringer et al., 2018).

Although the above-described modifications in the brain activity of various regions may facilitate motor learning, the effects of neuromodulation on such learning remain in dispute. Previous studies have shown that applying tACS to gamma band activity on the primary motor cortex or cerebellum is effective in motor learning (Bologna et al., 2019; Miyaguchi et al., 2019); meanwhile, others have shown no effect in similar interventions (Wessel et al., 2020). Therefore, consistent beneficial effects have not been established. One reason is that the characteristics of the EEG power spectra (the characteristics of neural oscillations) in the motor learning process are not clearly understood. Thereby, the appropriate frequency band and stimulation regions for neuromodulation interventions have not been determined. In order to develop effective intervention methods, it is necessary to reveal the characteristics of the power spectra during motor learning and to consider the frequency band and stimulation region to be modulated.

In the present study, to better understand the characteristics of the EEG power spectra during the proficiency process of hand skills for construction of an effective neuromodulation method, we measured the EEG of healthy participants during a hand motor learning task. We hypothesized that neural oscillation changes would occur in regions related to motor and sensory information processing that contribute to the motor learning in EEG measurements of the whole brain. By comparing the EEG activity of a motor learning task and a similar task without motor learning, we sought to verify this hypothesis. We used a task commonly used in studies of motor learning tasks—namely, the rotation of two balls on the palm—and we divided this task into a learning task and non-learning task by changing the direction of rotation, and compared the differences between the two tasks.

## 2. Methods

### 2.1. Participants

Fifteen healthy participants were included in the experiment (5 female; mean age = 22.8 years; standard deviation = 3.0). The sample size was determined based on previous studies (Manganotti et al., 1998; Shibata et al., 2021) and on behavioral data (repeated measures between the session and the direction of rotation in the number of rotations of the motor task described below; alpha = 0.05, power = 0.95, effect size = 0.35) using the G*Power software (Faul et al., 2009). The inclusion criteria were right-handedness according to the Edinburgh handedness inventory (median score 90, 25th percentile: 80; 75th percentile: 100) and no history of orthopedic or neurological disease in the left arm or hand. The experiment was approved by the ethics committee of the Faculty of Engineering at the University of Tokyo (approval number: KE20-21). All procedures were conducted in accordance with the standards set out in the World Medical Association Declaration of Helsinki. Written informed consent was obtained from all participants.

### 2.2. Procedure

The motor task was a ball-rotation task in which two balls were rotated in the palm of the left hand (Kawasaki et al., 2015, 2019). The balls (Uchida-denshi, Hachioji, Japan) were made from resin and were 50 mm in diameter, weighed 60 g each, and had a smooth surface. The task was an unskilled motor task that participants had not performed before. We selected this motor task because it involves visual and somatosensory input in the initial stages, and the re-weighting of these sensory information changes over time. The processes are relevant to learning in rehabilitation.

**Figure 1:**
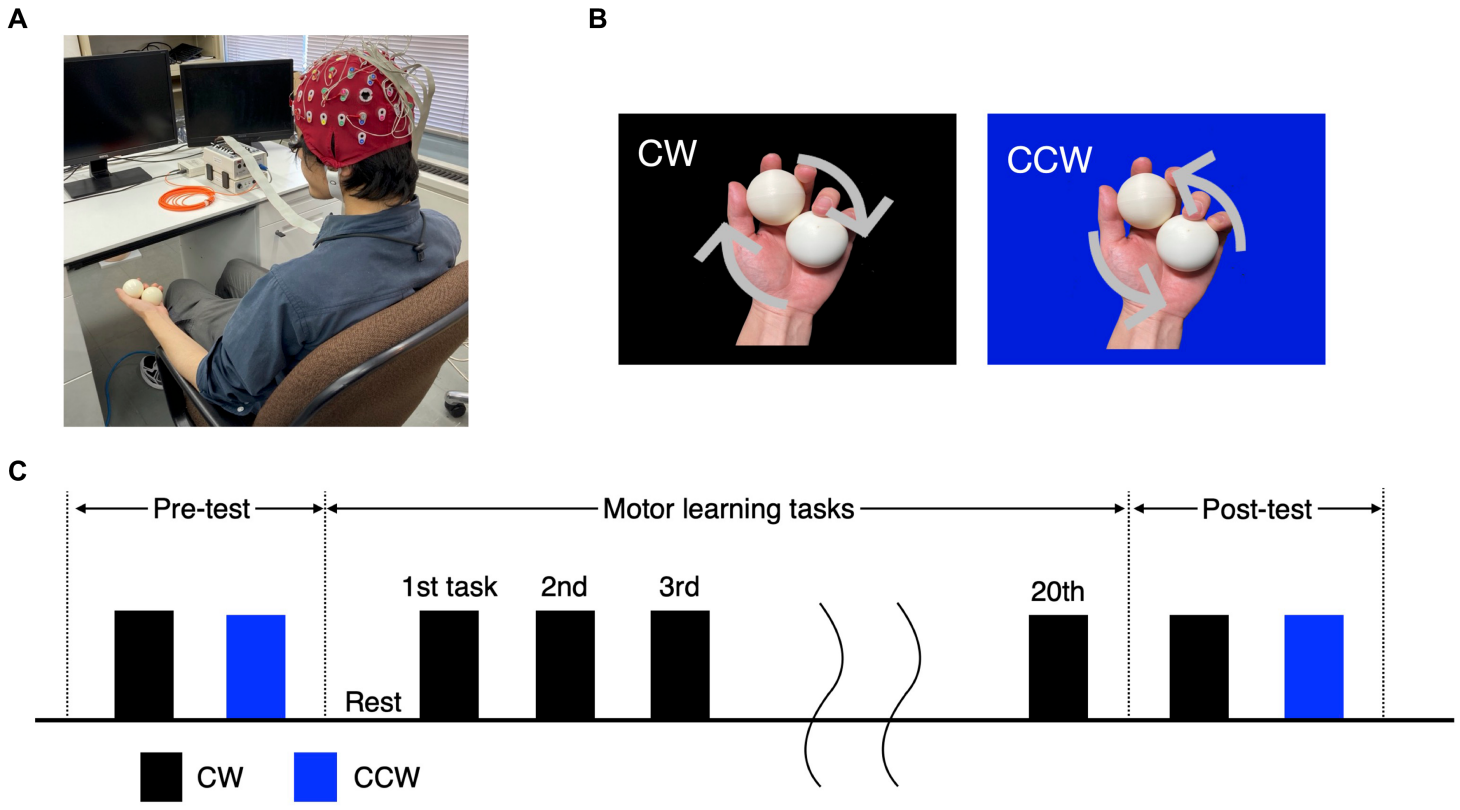
Experimental tasks and protocol. **(A)** Experimental environment and participants’ posture. **(B)** The ball-rotation tasks. The CW and CCW indicate the direction of clockwise and counterclockwise (control task), respectively. **(C)** The protocol of the experiment. In the pre-/post-test, the CW and CCW were performed once each, and the order of trials was randomized for each participant. The motor learning task was performed as 20 trials in the CW only.

Participants sat comfortably in a chair with armrests (height from seat: approximately 18 cm) and conducted a practice session, pre-test, motor learning task and post-test (Figure 1A). The practice session consisted of 10 clockwise (CW) and 10 counterclockwise (CCW) rotations (Figure 1B), and it was confirmed that the tasks were feasible. In the motor learning task, participants performed the CW task for 60 s per trial, for a total of 20 trials (Figure 1C). In the pre-test and post-test, participants performed one trial each of the CW and CCW (control condition) tasks for 60 s. The order of the CW and CCW in the pre/post-tests was randomized for each participant.

Behavioral data of the number of rotations per 60 s were recorded for all trials of the pre-test, motor learning task, and post-test. Participants were instructed to rotate the balls as quickly as possible during all trials, while taking care not to drop them. During all trials, participants were instructed to gaze at their left hand while executing the motor task in order to minimize artifacts of the electrooculogram (EOG). If participants dropped a ball, the relevant trial was terminated and a new trial was added. The number of rotations was recorded using a video camera (iVIS HF R42; Canon) during all sessions and calculated by experimenters after the experiment.

A trial consisted of rest (15 s), preparation (15 s) and motor task (60 s). For the rest, participants were asked to relax and told that they could move their gaze freely. For the preparation, participants were asked to gaze at their left hand in the same posture as in the motor task. Furthermore, participants could take a few minutes of rest after every 5 trials. During rest periods, the participants were requested to minimize sudden or excessive neck movements to prevent putting tension on the EEG cables, but were permitted to move their necks within reasonable limits. The beginning and end of the preparation and motor task were indicated by auditory stimuli (a beep at 750 Hz, duration 700 msec) using Psychtoolbox-3 (Brainard, 1997; Pelli, 1997; Kleiner et al., 2007) running on Matlab 2020a (Mathworks, Natick, MA).

### 2.3. EEG recording

EEG activity was recorded throughout each trial using an electroencephalograph (Active Two System, Biosemi) with a total of 32 electrodes (Fp1, Fp2, AFz, Fz, F3, F4, F7, F8, FCz, FC1, FC2, FC3, FC4, Cz, C3, C4, C5, C6, CPz, CP1, CP2, CP3, CP4, CP5, CP6, Pz, P3, P4, P7, P8, POz and Oz) and using the international 10–20 method. Electrodes were positioned according to traditional methods, using head caps adapted to the head size of each participant. Additionally, reference electrodes were placed on the bilateral mastoid, and left and right EOG (4 electrodes; SO1, LO1, LO2 and IO1, which reflect placements on the left superior orbit, left and right canthi and left inferior orbit respectively) were recorded to identify muscle activity during eye blinks that were included in the EEG. The value of the voltage offset was set to a level below 30 mV, confirming that the impedance of each electrode was reduced. The sampling frequency was set at 2,048 Hz. The data for 10 s after the start and 10 s before the end of each trial were excluded from the 60 s data for each trial, and 40 s was used as the analysis interval.

Pre-processing and power spectra analysis of EEG data in all trials were performed using the open source MATLAB toolbox EEGLab v2020.0 (Delorme and Makeig, 2004). The obtained raw EEG data were first offline re-referenced to the average signal across all electrodes, bandpass filtered between 1 and 80 Hz, and additionally filtered with a 50 Hz notch filter to reduce line noise artifacts. The data from each 40 s of all trials was divided into 5 s epochs, and epochs with artifacts were excluded by visual inspection and EEGLab’s statistical inspection regarding the abnormal values, trends, distributions, and improbable data from the analysis. Independent components analysis was conducted, and components involving eye blink or other muscle contraction artifacts recorded by EEG and EOG electrodes distinguished from brain-originated signals were subtracted based on amplitude and frequency characteristics.

The band-power was then computed to compare power spectra in all trials. A discrete Fast Fourier Transform with the Welch method was used in each epoch as the calculation method. The frequency range was divided into alpha (8–13 Hz), beta (13–30 Hz), low gamma (30–50 Hz), and high gamma (50–80 Hz) bands. The frequency ranges were determined based on previous studies (Sarnthein et al., 2006; Tatti et al., 2021).

### 2.4. EMG recording

Electromyographic (EMG) activities were measured to account for the influence of EEG activity in the motor cortex resulting from increased muscle activity in the pre-/post-tests. A wireless surface EMG sensor (Cometa Corp.) was used for the measurements. The abductor pollicis brevis muscle (APB) and the first dorsal interosseous muscle (FDI) involved in the ball-rotation task were selected as the muscles to be measured, based on a previous study (Aoyama and Kohno, 2020). The sampling frequency was set at 2,000 Hz. The data for 10 s after the start and 10 s before the end of each trial were excluded from the 60 s data for each trial, and 40 s was used as the analysis interval to analyze muscle activities in a psychologically stable state. Muscle activity was normalized with the maximum voluntary contraction (MVC; 5 s isometric contraction, three repetitions), which was measured before the pre-test.

The measured EMG signals were first band-pass filtered from 40–400 Hz with a 4th-order Butterworth digital filter, to debilitate DC offset and high-frequency noise. The filtered EMGs were then rectified and low-pass filtered (2nd order, cut-off frequency 5 Hz). As in the previous study (Aoyama and Kohno, 2020), the values above 5% of MVC during each test were defined as the interval in which muscle activity occurred, and the total EMG area as amount of muscle work (sum areas under the curve) was calculated. All pre-processing and calculations of the EMG area were conducted using Matlab 2020a.

### 2.5. Statistical analysis

The number of rotations was compared between the session (pre-/post-test) and the direction of rotation (CW/CCW) using a two-way repeated measures analysis of variance (ANOVA). The level of significance was set to *p* < 0.05. If an interaction effect was significant, paired *t*-test was conducted as a post-hoc test (four comparisons: pre-test CW vs. pre-test CCW; pre-test CW vs. post-test CW; pre-test CCW vs. post-test CCW; and post-test CW vs. post-test CCW) with Bonferroni correction after confirming the normality of the data using the Shapiro–Wilk test. The level of significance was set to *p* < 0.013. The level of significance for all statistical tests was set to *p* < 0.05. The areas of APB and FDI muscles in the CW were compared between the pre-/post-tests using a two-way repeated measures ANOVA. All data were analyzed using IBM SPSS Statistics 28. For the effect size of the behavioral data, values were calculated as *r* value.

In the analysis of EEG signals, a nonparametric method of permutation test was used to compare pre-test and post-test data, after rejecting the normality of some data using the Shapiro–Wilk test. The significance level was *p* < 0.01 (uncorrected) based on a previous study in an effort to prevent α errors (Perfetti et al., 2011; Daitch et al., 2013). In addition, a conservative method of adjustment was performed using the Bonferroni correction. Furthermore, in order to reveal the relation between the behavioral data and absolute power values in the locations that showed significantly increased power spectra in the post-test, Pearson correlation coefficient with an FDR correction was used. The data were analyzed using the mean values (the number of rotations and the absolute power value in each trial) of all participants. The significance level was *p* < 0.05.

Additionally, dipole localization estimation was performed to identify signal sources in regions where EEG analysis showed a significant increase in activity between the pre-test and post-test. The EEGLab tool DIPFIT2 was used to obtain Talairach coordinates of the averaged locations of the clusters of independent components involved in the task. The obtained coordinates were identified as the corresponding brain region (Brodmann Area) using MNI2Tal^1^ and Talairach client (Lancaster et al., 2000).

## 3. Results

### 3.1. Behavioral changes in the pre-/post-tests

**Figure 2:**
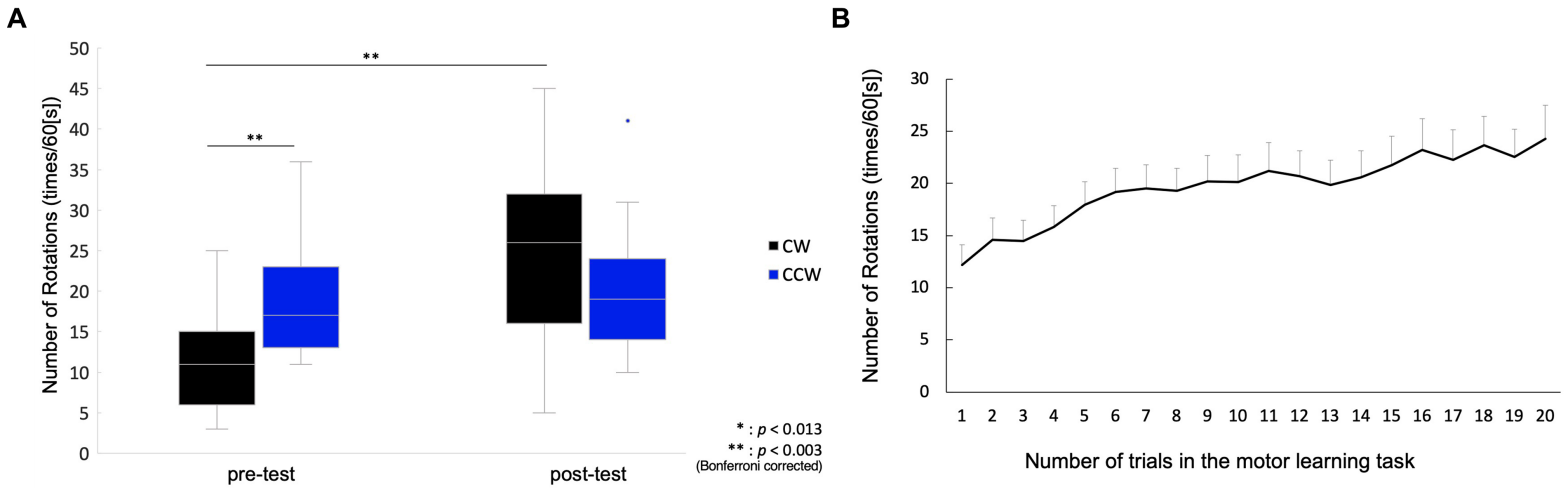
Behavioral change between pre-/post-tests, and in motor learning process. **(A)** Box plot for the number of rotations in the pre-/post-tests. The middle horizontal line in the box represents the median. The bottom and top of the box indicate the first and third quartiles, respectively. The vertical lines extend from the minimum to the maximum value. A dot indicates an outlier. **(B)** Mean values and standard error for the number of rotations in the motor learning task. The plot shows changes in the number of rotations from the 1st to 20th trial.

In the pre-test, the number of rotations of the CW was 11.6 ± 1.9 (mean ± standard error), and that of the CCW was 18.5 ± 1.5. In the post-test, the number of rotations in the CW was 25.5 ± 2.6 and that in the CCW was 19.3 ± 2.2. Two-way repeated measures ANOVA showed a significant main effect of condition (pre-/post-test) (*F*(1, 14) = 20.88, *p* < 0.001), but no significant main effect of rotation direction (CW/CCW) (*F*(1, 14) = 0.37, *p* = 0.554). In addition, an interaction effect was observed (*F*(1, 14) = 37.96, *p* < 0.001), and the post-hoc test showed significant differences between the pre-test CW and pre-test CCW [*t*(1, 14) = 3.94, *p* = 0.001], and between the pre-test CW and post-test CW [*t*(1, 14) =6.13, *p* < 0.001], but there was no significant difference between the post-test CW and post-test CCW [*t*(1, 14) = 2.50, *p* = 0.025], or between the pre-test CCW and post-test CCW [*t*(1, 14) = 1.12, *p* = 0.282] (Figure 2A). The mean of the number of rotations in each trial during the motor learning task is shown in Figure 2B. The number of drops in all motor learning tasks was 2.1 ± 0.4 (mean ± standard error) for all participants.

**Figure 3:**
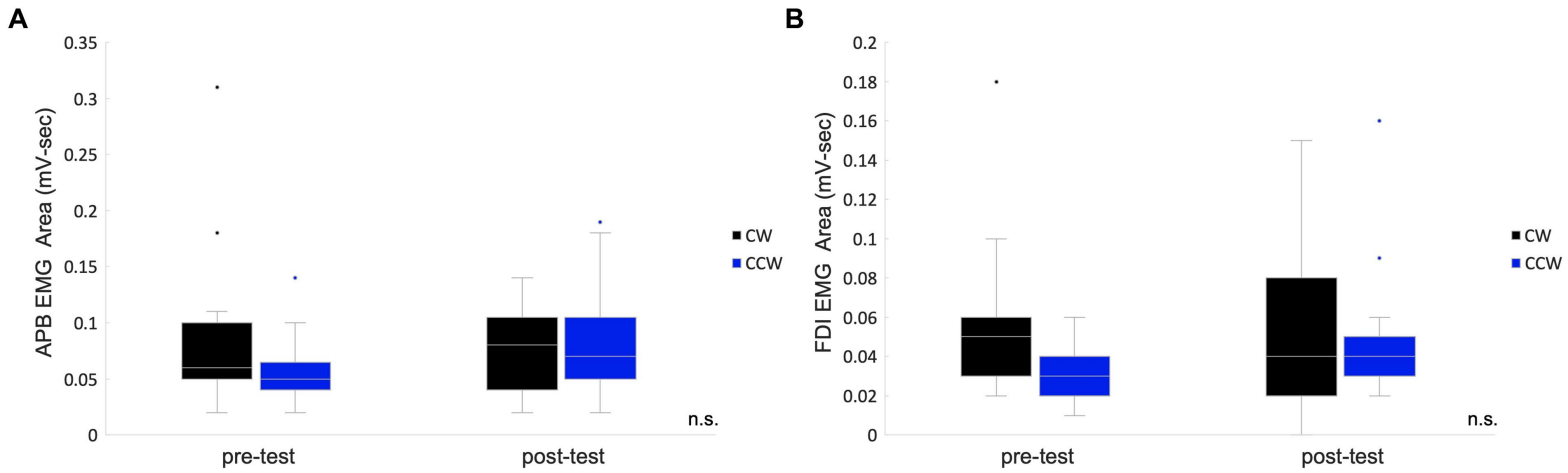
Box plots for the EMG areas in the pre-/post-tests. The values of the total muscle activity area in the pre-/post-tests are shown in box plots which indicate the area of the abductor pollicis brevis muscle **(A)** and the first dorsal interosseous muscle. **(B)** The middle horizontal line represents the median. The bottom and top of the box indicate the first and third quartiles, respectively. The vertical lines extend from the minimum to the maximum value. A dot indicates an outlier. n.s., not significant.

The EMG area of the APB muscle of CW was 0.09 ± 0.02 mV-sec (mean ± standard error), and that of the CCW was 0.06 ± 0.01 mV-sec in the pre-test. The APB muscle of CW was 0.08 ± 0.01 mV-sec, and that of the CCW was 0.08 ± 0.01 mV-sec in the post-test. In the analysis of APB, data from two participants were excluded due to problems with electrode contact. The FDI muscle of CW was 0.06 ± 0.01 mV-sec and that of the CCW was 0.03 ± 0.00 mV-sec in the pre-test. The FDI muscle of CW was 0.05 ± 0.01 mV-sec and that of the CCW was 0.05 ± 0.01 mV-sec in the post-test. Two-way repeated measures ANOVA showed no significant main effects of condition (pre-/post-test) (APB: *F*(1,12) = 0.42, *p* = 0.531, FDI: *F*(1,14) = 1.65, *p* = 0.220), main effects of rotation direction (APB: *F*(1,12) = 0.40, *p* = 0.539, FDI: *F*(1,14) = 4.43, *p* = 0.054) and interaction effects (APB: *F*(1,12) = 3.54, *p* = 0.085, FDI: *F*(1,14) = 0.70, *p* = 0.418) (Figure 3).

### 3.2. EEG changes between pre-/post-test

**Figure 4:**
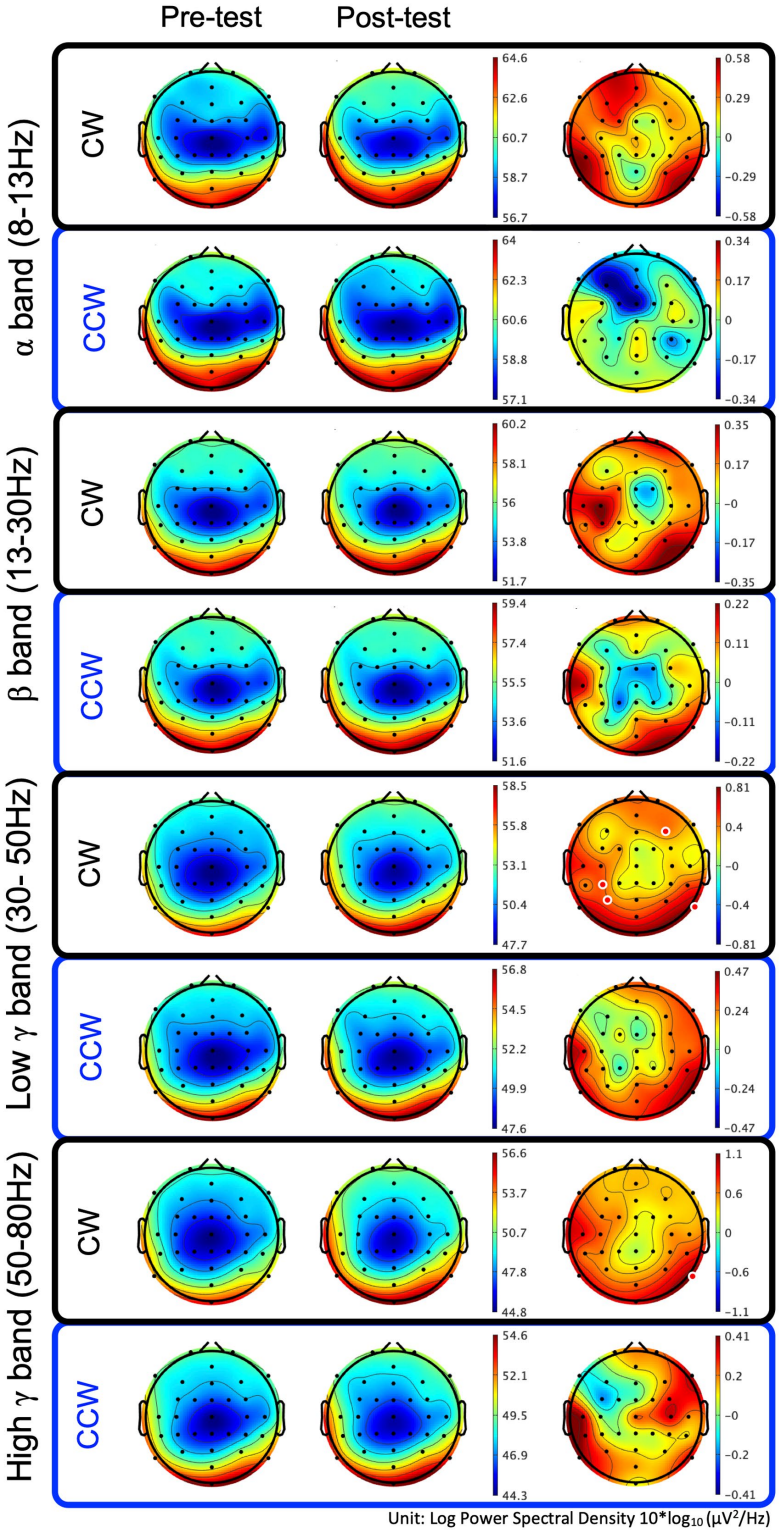
Brain topographic maps of the alpha, beta, and low−/high-gamma bands and spectral change with significant differences between the pre-/post-tests. The two rows at left show brain topographic maps in the pre-test and post-test. The right-hand row shows the spectral change (red regions in the color map represent power higher than the averaged power of the pre-/post-test) and locations with significant differences between the pre-/post-tests. The red points in the color map in the right row indicate the regions with significant differences (*p* < 0.01, uncorrected). CW and CCW indicate the clockwise and counterclockwise (control task) directions, respectively. Note that each scaling of the power spectral values is different.

**Figure 5:**
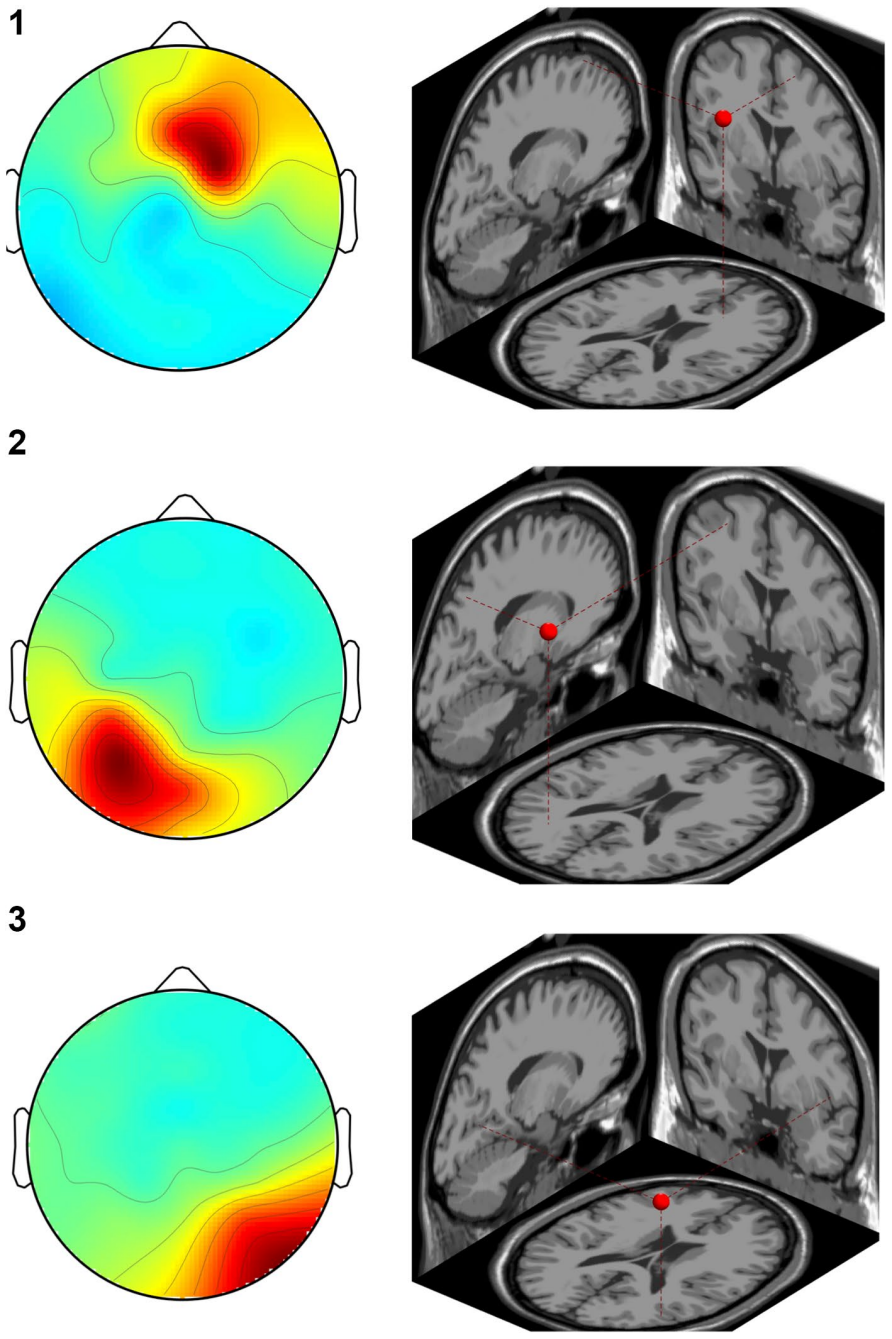
Representative clusters in the right frontal region, left parietal region and right temporal region identified by dipole estimation. The regions of interest were identified as regions with significant differences in the gamma bands, and the signal sources were identified by dipole estimation. The numbers correspond to the cluster number in Table 2.

In the CW, there were no significant differences in the alpha and beta bands (*p* > 0.01). In the low gamma band, there were significant differences in F4, CP3, P3 and P8 (*p* < 0.01) (Figure 4; Table 1) which increased in the post-test. In the high gamma band, there was significant difference in P8 (*p* < 0.01) (Figure 4; Table 1) which increased in the post-test. The averaged locations of the representative clusters of independent components were observed in the frontal region (right premotor area and supplemental motor area), left parietal region and right temporal region, as shown in Figure 5 and Table 2. There were no significant differences in the corrected *p*-values for all frequency bands.

**Table 1:** Locations with significant differences in power spectra between the pre-/post-test.

	Locations	Pre-test	Post-test	*p* value
Low gamma	F4	0.85 (0.15)	0.97 (0.14)	< 0.01	CP3	0.58 (0.08)	0.85 (0.25)	< 0.01	P3	0.81 (0.10)	1.04 (0.14)	< 0.01	P8	2.13 (0.38)	3.49 (0.85)	< 0.01
High gamma	P8	2.08 (0.45)	3.61 (0.93)	< 0.01

Unit: μV^2^, values are the average, values in parenthesis are the standard error; Permutation test: *p* < 0.01 (uncorrected).

**Table 2:** Location of the regions of independent component clusters.

Cluster number	Coordinates			Region	BA
	*x*	*y*	*z*		
1	19	29	54	Right superior frontal gyrus	6
2	−29	−60	−50	Left superior parietal lobule	7
3	47	−62	8	Middle temporal gyrus	37

Coordinates in Talairach space; BA, Brodmann area.

In the CCW, there were no significant differences in any of the frequency bands (uncorrected and corrected).

### 3.3. Correlation between the number of rotations and the EEG power values in each trial during the motor learning process

**Figure 6:**
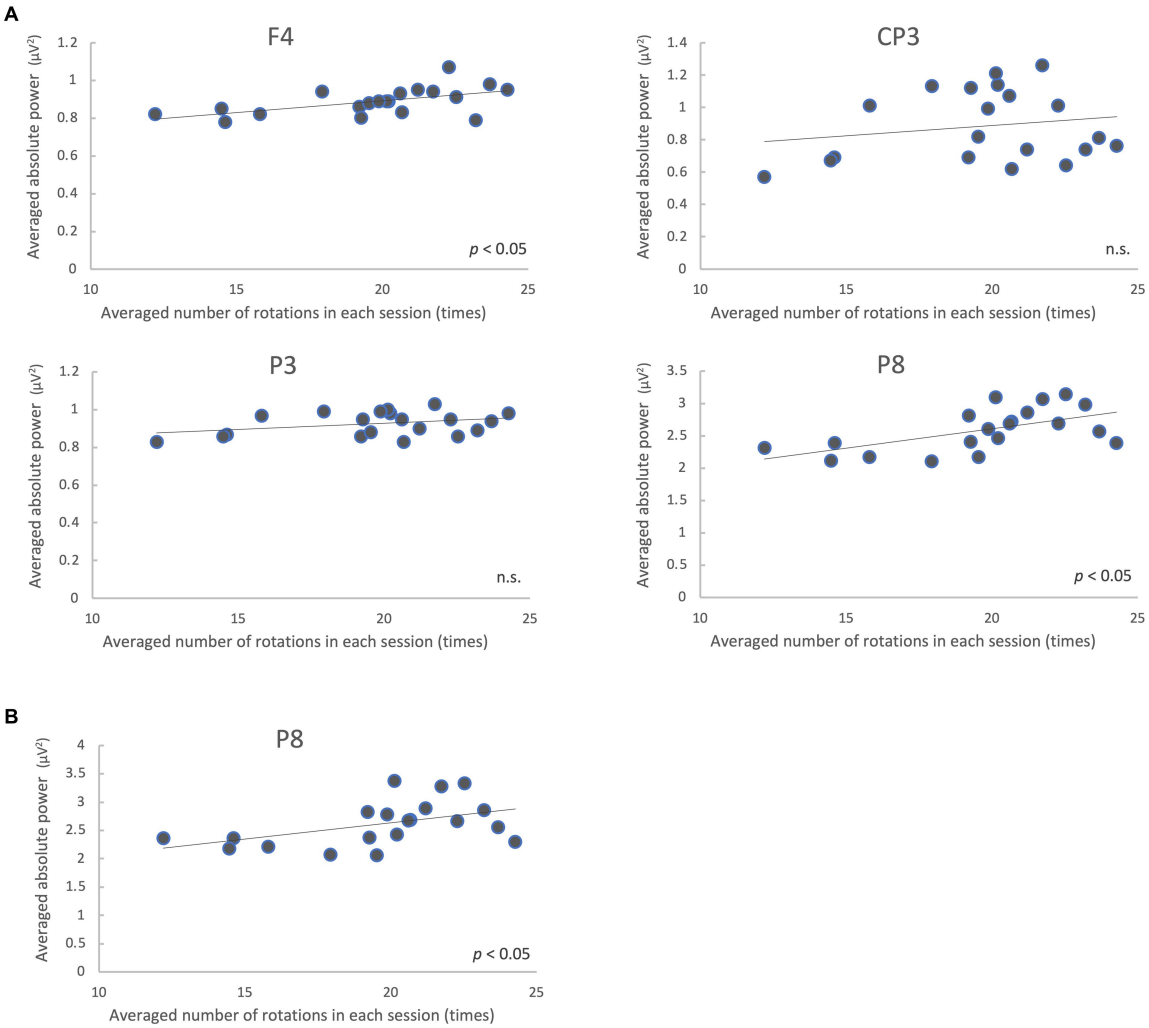
Correlation between the mean of all participants’ number of rotations and EEG absolute power values on each trial during the motor learning task. Absolute power values in the low gamma band **(A)** and high gamma band **(B)** are shown. n.s., not significant.

We analyzed the correlation between the mean of all participants’ number of rotations and the mean of the absolute power values on each trial in the regions where the power spectra showed significant differences (low gamma band: 4 regions; high gamma band: 1 region) between the pre-/post-tests. The results showed that F4 and P8 were significantly correlated (*p* < 0.05, FDR-corrected) in the low gamma band, and P8 was significantly correlated in the high gamma band (*p* < 0.05) (Figure 6; Table 3).

**Table 3:** Correlation between the number of rotations and EEG absolute power values during the motor learning task.

	Locations	Correlation coefficient (*r*)	*p* value
Low gamma	F4	0.55	0.01 *	CP3	0.19	0.42	P3	0.34	0.14	P8	0.59	< 0.01 *
High gamma	P8	0.47	0.03 *

Correlation coefficient (*r*), Pearson correlation coefficient; *, significant differences.

## 4. Discussion

In the present study, to understand the characteristics of the EEG power spectra related to motor learning, we recorded EEG activity before and after a motor learning task, as well as during the task. After a total of 20 min of motor learning, the performance of the motor task was enhanced, and the EEG power spectra showed increased activity in the low and high gamma bands in the right frontal, left lateral parietal, and right temporal regions. In addition, there were positive correlations between the right frontal and right temporal regions’ power spectra and the performance of the ball-rotation task in the motor learning process.

The gamma band is composed of neural oscillation by inhibitory interneurons (Whittington et al., 1995), and the control of inhibitory interneurons in inhibition and dis-inhibition regulates excitatory neurons. In general, increased power in the gamma band reflects a state of increased local cortical activity (Fries, 2015; Sohal, 2016), and thus the significant increase in the power spectra of the gamma band in the frontal, parietal, and right temporal regions observed in the present study suggests that each region was in a state of increased activity.

The gamma band power of the frontal region showed increased activity in FC4, which corresponds to the right premotor and supplemental motor areas (BA6) and is a region related to planning of movement. This region has particularly important implications for motor control (Schluter et al., 2001) and is also known to be involved in action selection and planning (Nakayama et al., 2022). In addition, the premotor area plays an important role in responding to immediate motor changes, influencing M1 to update the motor strategy during grasping (Buch et al., 2010). The motor task in the present study required the selection of movement and changes in motor strategy in accordance with the state of the ball, which could be considered to indicate an increase in activity in the regions involved in motor control.

On the other hand, a previous study reported that the gamma band of the primary motor cortex was activated in response to contralateral hand movements (Crone, 1998). In our findings, however, there was no increase in the activity of the primary motor cortex in the motor learning process. Although the primary motor cortex is involved in motor output, this area reflects the final output, and is probably not important in motor learning. In addition, the fact that there was no increase in EMG activity suggests that the change in motor pattern was more important than the increase in muscle activity as a characteristic of our experimental task. Therefore, it is inferred that no change in motor cortex activity was observed as opposed to the premotor cortex. In a review, Takeuchi and Izumi (Takeuchi and Izumi, 2021) reported that tACS interventions on the primary motor cortex only single site have no consistent effect on motor learning. Therefore, it can be inferred that the change in M1 activity is likely to be not mandatory in motor skill learning. Our results support the inference from the viewpoint of the EEG power spectra. We emphasize the necessity of interventions that comply with the EEG power spectra according to the characteristics of tasks.

Increased activity was observed in the gamma bands of the temporal region (P8). The right temporal region (fusiform gyrus) corresponds the extrastriate body area (EBA), which is a region that is specifically activated when looking at the body or body parts (Downing et al., 2001). It has been reported that pictures of human dynamic states increase EBA activity compared to pictures of static states (Proverbio et al., 2009), and that the EBA is involved in motor planning (Zimmermann et al., 2012, 2016). Therefore, it is likely that the visual attention paid to body parts and planning of physical activity for improving motor skills during the motor learning task affected the activity level of the EBA in the present study.

The left parietal area (CP3 and P3) corresponding the superior parietal lobule is known to be involved in skill acquisition related to sequential learning (Hikosaka et al., 2002) and in recognizing the location of body parts (Felician et al., 2004). The task in the present study involved multiple fingers, as well as a sequence to be learned in order to rotate the balls. However, it is difficult to fully examine the functional role played by this region during motor learning tasks, as no correlation was found between frequency power and performance during motor learning tasks.

The results of the present study suggest that tACS and tRNS interventions that modify gamma band oscillations may enhance the efficiency of newly learned hand dexterity. Specifically, as a precondition for motor output, neuromodulation interventions that consider motor planning and attention to body parts could be effective. The right frontal region is likely to be involved in the left-hand movement, while the parietal and temporal regions might be involved in multiple types of motor learning using other body parts. As areas for intervention, those could be worthy of consideration. Recent studies have demonstrated that paired stimulation with TMS can access the neural networks between areas (Veniero et al., 2013; Breveglieri et al., 2021). Repetitive TMS is a commonly used intervention technique for stroke patients (Fisicaro et al., 2019), and interventions using paired stimulation for frequency modifications are anticipated. Therefore, repetitive TMS may also lead to EEG-based interventions that could be used together with tACS and tRNS to elucidate the frequency characteristics in the whole brain.

The present study has several limitations. First, only one task was selected as a motor learning task. The intensity of the EEG power spectra and the regions of activity may change due to changes in body parts (differences between left and right, upper and lower limbs) and the characteristics of the motor task. In particular, it is likely that increased activity in the contralateral hemisphere is inferred for the frontal regions related to movements, and changes in activity in the left hemisphere are inferred for right-handed tasks. Therefore, it is necessary to understand the characteristics of the EEG power spectra through further investigation, considering the characteristics of the motor task and body parts involved. Additionally, the locations of the electrodes are not digitized by MRI or other methods, and the small number of electrodes is a limitation in accurately indicating the location of the signal. In order to establish a strong basis for the study, additional validation is required.

## 5. Conclusion

The present study revealed the characteristics of EEG power spectral changes during motor learning of the left hand. In particular, increased power spectra in the low and high gamma band were observed in frontal, parietal, and temporal areas, and these bands and areas provide a point of focus for neuromodulation interventions. Based on these findings, further research is needed to verify the effects of interventions and develop neuromodulation techniques to modulate motor learning in healthy persons, so as to eventually lead to recovery in persons with neurodegenerative disease or stroke.

## Data availability statement

The raw data supporting the conclusions of this article will be made available by the authors, without undue reservation.

## Ethics statement

The studies involving human participants were reviewed and approved by the ethics committee of the Faculty of Engineering at the University of Tokyo. The patients/participants provided their written informed consent to participate in this study.

## Author contributions

HH: conception, investigation, and methodology of the study, data analysis, and drafting and revising of the manuscript. WW and TK: conception, investigation, and methodology of the study, and editing of the manuscript. AY and HA: conception and supervision of the study and editing and revising of the manuscript. All authors contributed to the article and approved the submitted version.

## Funding

This work was supported by JSPS KAKENHI Grant Numbers JP19H05729 and JP20K19423. WW was additionally supported by JST FOREST Program, Grant Number JPMJFR2144.

## Conflict of interest

The authors declare that the research was conducted in the absence of any commercial or financial relationships that could be construed as a potential conflict of interest.

## Publisher’s note

All claims expressed in this article are solely those of the authors and do not necessarily represent those of their affiliated organizations, or those of the publisher, the editors and the reviewers. Any product that may be evaluated in this article, or claim that may be made by its manufacturer, is not guaranteed or endorsed by the publisher.

## Footnotes

^1^ https://bioimagesuiteweb.github.io/webapp/mni2tal.html

## References

[ref1] Antal A. Boros K. Poreisz C. Chaieb L. Terney D. Paulus W. (2008). Comparatively weak after-effects of transcranial alternating current stimulation (tACS) on cortical excitability in humans. Brain Stimul. 1, 97–105. doi: 10.1016/j.brs.2007.10.001, PMID: 20633376

[ref2] Antal A. Herrmann C. S. (2016). Transcranial alternating current and random noise stimulation: possible mechanisms. Neural Plast. 2016, 3616807–3616812. doi: 10.1155/2016/3616807, PMID: 27242932 PMC4868897

[ref3] Aoyama T. Kohno Y. (2020). Temporal and quantitative variability in muscle electrical activity decreases as dexterous hand motor skills are learned. PLoS One 15:e0236254. doi: 10.1371/journal.pone.0236254, PMID: 32687520 PMC7371173

[ref4] Bologna M. Guerra A. Paparella G. Colella D. Borrelli A. Suppa A.. (2019). Transcranial alternating current stimulation has frequency-dependent effects on motor learning in healthy humans. Neuroscience 411, 130–139. doi: 10.1016/j.neuroscience.2019.05.041 31152934

[ref5] Brainard D. H. (1997). The psychophysics toolbox. Spat. Vis. 10, 433–436. doi: 10.1163/156856897X00357 9176952

[ref6] Breveglieri R. Borgomaneri S. Filippini M. De Vitis M. Tessari A. Fattori P. (2021). Functional connectivity at rest between the human medial posterior parietal cortex and the primary motor cortex detected by paired-pulse transcranial magnetic stimulation. Brain Sci. 11:1357. doi: 10.3390/brainsci11101357, PMID: 34679421 PMC8534070

[ref7] Buch E. R. Mars R. B. Boorman E. D. Rushworth M. F. S. (2010). A network centered on ventral premotor cortex exerts both facilitatory and inhibitory control over primary motor cortex during action reprogramming. J. Neurosci. 30, 1395–1401. doi: 10.1523/JNEUROSCI.4882-09.2010, PMID: 20107065 PMC2880444

[ref8] Crone N. (1998). Functional mapping of human sensorimotor cortex with electrocorticographic spectral analysis. II. Event-related synchronization in the gamma band. Brain 121, 2301–2315. doi: 10.1093/brain/121.12.2301, PMID: 9874481

[ref9] Daitch A. L. Sharma M. Roland J. L. Astafiev S. V. Bundy D. T. Gaona C. M.. (2013). Frequency-specific mechanism links human brain networks for spatial attention. Proc. Natl. Acad. Sci. U. S. A. 110, 19585–19590. doi: 10.1073/pnas.1307947110, PMID: 24218604 PMC3845177

[ref10] Delorme A. Makeig S. (2004). EEGLAB: an open source toolbox for analysis of single-trial EEG dynamics including independent component analysis. J. Neurosci. Methods 134, 9–21. doi: 10.1016/j.jneumeth.2003.10.009, PMID: 15102499

[ref11] Downing P. E. Jiang Y. Shuman M. Kanwisher N. (2001). A cortical area selective for visual processing of the human body. Science 293, 2470–2473. doi: 10.1126/science.1063414 11577239

[ref12] Eberle H. Hayashi Y. Kurazume R. Takei T. An Q. (2021). Modeling of hyper-adaptability: from motor coordination to rehabilitation. Adv. Robot. 35, 802–817. doi: 10.1080/01691864.2021.1943710

[ref13] Faul F. Erdfelder E. Buchner A. Lang A.-G. (2009). Statistical power analyses using G*power 3.1: tests for correlation and regression analyses. Behav. Res. Methods 41, 1149–1160. doi: 10.3758/brm.41.4.1149 19897823

[ref14] Felician O. Romaiguère P. Anton J.-L. Nazarian B. Roth M. Poncet M.. (2004). The role of human left superior parietal lobule in body part localization. Ann. Neurol. 55, 749–751. doi: 10.1002/ana.20109, PMID: 15122719

[ref15] Fisicaro F. Lanza G. Grasso A. A. Pennisi G. Bella R. Paulus W.. (2019). Repetitive transcranial magnetic stimulation in stroke rehabilitation: review of the current evidence and pitfalls. Ther. Adv. Neurol. Disord. 12:1756286419878317. doi: 10.1177/1756286419878317, PMID: 31598137 PMC6763938

[ref16] Fries P. (2015). Rhythms for cognition: communication through coherence. Neuron 88, 220–235. doi: 10.1016/j.neuron.2015.09.034, PMID: 26447583 PMC4605134

[ref17] Gehringer J. E. Arpin D. J. Heinrichs-Graham E. Wilson T. W. Kurz M. J. (2018). Neurophysiological changes in the visuomotor network after practicing a motor task. J. Neurophysiol. 120, 239–249. doi: 10.1152/jn.00020.2018, PMID: 29589817 PMC6093962

[ref18] Herrmann C. S. Rach S. Neuling T. Strüber D. (2013). Transcranial alternating current stimulation: a review of the underlying mechanisms and modulation of cognitive processes. Front. Hum. Neurosci. 7:279. doi: 10.3389/fnhum.2013.00279, PMID: 23785325 PMC3682121

[ref19] Hikosaka O. Nakamura K. Sakai K. Nakahara H. (2002). Central mechanisms of motor skill learning. Curr. Opin. Neurobiol. 12, 217–222. doi: 10.1016/s0959-4388(02)00307-0 12015240

[ref20] Hsieh Y.-W. Lee M.-T. Lin Y.-H. Chuang L.-L. Chen C.-C. Cheng C.-H. (2021). Motor cortical activity during observing a video of real hand movements versus computer graphic hand movements: an MEG study. Brain Sci. 11:6. doi: 10.3390/brainsci11010006, PMID: 33374670 PMC7822490

[ref21] Kang N. Summers J. J. Cauraugh J. H. (2016). Transcranial direct current stimulation facilitates motor learning post-stroke: a systematic review and meta-analysis. J. Neurol. Neurosurg. Psychiatry 87, 345–355. doi: 10.1136/jnnp-2015-311242, PMID: 26319437

[ref22] Kawasaki T. Aramaki H. Tozawa R. (2015). An effective model for observational learning to improve novel motor performance. J. Phys. Ther. Sci. 27, 3829–3832. doi: 10.1589/jpts.27.3829, PMID: 26834362 PMC4713801

[ref23] Kawasaki T. Kono M. Tozawa R. (2019). Efficacy of verbally describing one’s own body movement in motor skill acquisition. Brain Sci. 9:356. doi: 10.3390/brainsci9120356, PMID: 31817257 PMC6956347

[ref24] Kim S. Ogawa K. Lv J. Schweighofer N. Imamizu H. (2015). Neural substrates related to motor memory with multiple timescales in sensorimotor adaptation. PLoS Biol. 13:e1002312. doi: 10.1371/journal.pbio.1002312, PMID: 26645916 PMC4672877

[ref25] Kleiner M. Brainard D. Pelli D. Ingling A. Murray R. Broussard C. (2007). What is new in psychtoolbox 3. Perception 36, 1–16.

[ref26] Krakauer J. W. (2006). Motor learning: its relevance to stroke recovery and neurorehabilitation. Curr. Opin. Neurol. 19, 84–90. doi: 10.1097/01.wco.0000200544.29915.cc 16415682

[ref27] Lancaster J. L. Woldorff M. G. Parsons L. M. Liotti M. Freitas C. S. Rainey L.. (2000). Automated Talairach atlas labels for functional brain mapping. Hum. Brain Mapp. 10, 120–131. doi: 10.1002/1097-0193(200007)10:3<120::aid-hbm30>3.0.co;2-8 10912591 PMC6871915

[ref28] Lee K. J. Park I. S. Kim H. Greenough W. T. Pak D. T. S. Rhyu I. J. (2013). Motor skill training induces coordinated strengthening and weakening between neighboring synapses. J. Neurosci. 33, 9794–9799. doi: 10.1523/JNEUROSCI.0848-12.2013, PMID: 23739975 PMC3865495

[ref29] Macintosh B. J. Mraz R. McIlroy W. E. Graham S. J. (2007). Brain activity during a motor learning task: an fMRI and skin conductance study. Hum. Brain Mapp. 28, 1359–1367. doi: 10.1002/hbm.20351, PMID: 17318835 PMC4896816

[ref30] Manganotti P. Gerloff C. Toro C. Katsuta H. Sadato N. Zhuang P.. (1998). Task-related coherence and task-related spectral power changes during sequential finger movements. Electroencephalogr. Clin. Neurophysiol. 109, 50–62. doi: 10.1016/s0924-980x(97)00074-x, PMID: 11003064

[ref31] Manuel A. L. Guggisberg A. G. Thézé R. Turri F. Schnider A. (2018). Resting-state connectivity predicts visuo-motor skill learning. NeuroImage 176, 446–453. doi: 10.1016/j.neuroimage.2018.05.003, PMID: 29730496

[ref32] Miyaguchi S. Otsuru N. Kojima S. Yokota H. Saito K. Inukai Y.. (2019). Gamma tACS over M1 and cerebellar hemisphere improves motor performance in a phase-specific manner. Neurosci. Lett. 694, 64–68. doi: 10.1016/j.neulet.2018.11.015, PMID: 30445151

[ref33] Mutha P. K. Sainburg R. L. Haaland K. Y. (2011). Left parietal regions are critical for adaptive visuomotor control. J. Neurosci. 31, 6972–6981. doi: 10.1523/JNEUROSCI.6432-10.2011, PMID: 21562259 PMC3107546

[ref34] Nakano H. Osumi M. Ueta K. Kodama T. Morioka S. (2013). Changes in electroencephalographic activity during observation, preparation, and execution of a motor learning task. Int. J. Neurosci. 123, 866–875. doi: 10.3109/00207454.2013.813509, PMID: 23768018

[ref35] Nakayama Y. Sugawara S. K. Fukunaga M. Hamano Y. H. Sadato N. Nishimura Y. (2022). The dorsal premotor cortex encodes the step-by-step planning processes for goal-directed motor behavior in humans. NeuroImage 256:119221. doi: 10.1016/j.neuroimage.2022.119221, PMID: 35447355

[ref36] Nowak M. Zich C. Stagg C. J. (2018). Motor cortical gamma oscillations: what have we learnt and where are we headed? Curr. Behav. Neurosci. Rep. 5, 136–142. doi: 10.1007/s40473-018-0151-z, PMID: 29862162 PMC5962618

[ref37] Özdenizci O. Yalçın M. Erdoğan A. Patoğlu V. Grosse-Wentrup M. Çetin M. (2017). Electroencephalographic identifiers of motor adaptation learning. J. Neural Eng. 14:046027. doi: 10.1088/1741-2552/aa6abd, PMID: 28367834

[ref38] Pelli D. G. (1997). The VideoToolbox software for visual psychophysics: transforming numbers into movies. Spat. Vis. 10, 437–442. doi: 10.1163/156856897X00366, PMID: 9176953

[ref39] Perfetti B. Moisello C. Landsness E. C. Kvint S. Lanzafame S. Onofrj M.. (2011). Modulation of gamma and theta spectral amplitude and phase synchronization is associated with the development of visuo-motor learning. J. Neurosci. 31, 14810–14819. doi: 10.1523/JNEUROSCI.1319-11.2011, PMID: 21994398 PMC3206224

[ref40] Proverbio A. M. Riva F. Zani A. (2009). Observation of static pictures of dynamic actions enhances the activity of movement-related brain areas. PLoS One 4:e5389. doi: 10.1371/journal.pone.0005389, PMID: 19421311 PMC2671843

[ref41] Sarnthein J. Stern J. Aufenberg C. Rousson V. Jeanmonod D. (2006). Increased EEG power and slowed dominant frequency in patients with neurogenic pain. Brain 129, 55–64. doi: 10.1093/brain/awh631, PMID: 16183660

[ref42] Schluter N. D. Krams M. Rushworth M. F. Passingham R. E. (2001). Cerebral dominance for action in the human brain: the selection of actions. Neuropsychologia 39, 105–113. doi: 10.1016/s0028-3932(00)00105-6 11163368

[ref43] Schmidt R. A. (1988). Motor control and learning: a behavioral emphasis. Champaign, IL: Human Kinetics.

[ref44] Shibata S. Watanabe T. Yukawa Y. Minakuchi M. Shimomura R. Ichimura S.. (2021). Effects of transcranial static magnetic stimulation over the primary motor cortex on local and network spontaneous electroencephalogram oscillations. Sci. Rep. 11:8261. doi: 10.1038/s41598-021-87746-2, PMID: 33859297 PMC8050201

[ref45] Sohal V. S. (2016). How close are we to understanding what (if anything) γ oscillations do in cortical circuits? J. Neurosci. 36, 10489–10495. doi: 10.1523/JNEUROSCI.0990-16.2016, PMID: 27733600 PMC5059424

[ref46] Takeuchi N. Izumi S. (2021). Motor learning based on oscillatory brain activity using transcranial alternating current stimulation: a review. Brain Sci. 11:1095. doi: 10.3390/brainsci11081095, PMID: 34439714 PMC8392205

[ref47] Tatti E. Ferraioli F. Peter J. Alalade T. Nelson A. B. Ricci S.. (2021). Frontal increase of beta modulation during the practice of a motor task is enhanced by visuomotor learning. Sci. Rep. 11:17441. doi: 10.1038/s41598-021-97004-0, PMID: 34465846 PMC8408223

[ref48] Veniero D. Ponzo V. Koch G. (2013). Paired associative stimulation enforces the communication between interconnected areas. J. Neurosci. 33, 13773–13783. doi: 10.1523/JNEUROSCI.1777-13.2013, PMID: 23966698 PMC6618662

[ref49] Wessel M. J. Draaisma L. R. de Boer A. F. W. Park C.-H. Maceira-Elvira P. Durand-Ruel M.. (2020). Cerebellar transcranial alternating current stimulation in the gamma range applied during the acquisition of a novel motor skill. Sci. Rep. 10:11217. doi: 10.1038/s41598-020-68028-9, PMID: 32641706 PMC7343806

[ref50] Whittington M. A. Traub R. D. Jefferys J. G. (1995). Synchronized oscillations in interneuron networks driven by metabotropic glutamate receptor activation. Nature 373, 612–615. doi: 10.1038/373612a0, PMID: 7854418

[ref51] Xu T. Yu X. Perlik A. J. Tobin W. F. Zweig J. A. Tennant K.. (2009). Rapid formation and selective stabilization of synapses for enduring motor memories. Nature 462, 915–919. doi: 10.1038/nature08389, PMID: 19946267 PMC2844762

[ref52] Zhu J.-D. Cheng C.-H. Tseng Y.-J. Chou C.-C. Chen C.-C. Hsieh Y.-W.. (2019). Modulation of motor cortical activities by action observation and execution in patients with stroke: an MEG study. Neural Plast. 2019, 8481371–8481310. doi: 10.1155/2019/8481371, PMID: 31781183 PMC6875039

[ref53] Zimmermann M. Meulenbroek R. G. J. de Lange F. P. (2012). Motor planning is facilitated by adopting an action’s goal posture: an fMRI study. Cereb. Cortex 22, 122–131. doi: 10.1093/cercor/bhr098, PMID: 21613471

[ref54] Zimmermann M. Verhagen L. de Lange F. P. Toni I. (2016). The extrastriate body area computes desired goal states during action planning. eNeuro 3:ENEURO.0020-16.2016. doi: 10.1523/ENEURO.0020-16.2016, PMID: 27066535 PMC4821904

